# Quantitative assessment of treatment efficacy in keloids using high-frequency ultrasound and shear wave elastography: a preliminary study

**DOI:** 10.1038/s41598-020-58209-x

**Published:** 2020-01-28

**Authors:** Song-Ya Huang, Xi Xiang, Rui-Qian Guo, Shan Cheng, Li-Yun Wang, Li Qiu

**Affiliations:** Department of Ultrasound, West China Hospital of Sichuan University, No. 37 Guo Xue Xiang, Chengdu, 610041 Sichuan Province China

**Keywords:** Diseases, Skin diseases

## Abstract

The purpose of this study was to investigate the performance of high-frequency ultrasound (HFUS) and shear wave elastography (SWE) in the quantitative evaluation of therapeutic responses of keloids. 43 patients with 76 keloids were recruited into this study. In keloids and symmetrical sites, the skin thickness was measured using HFUS and skin stiffness expressed as elastic moduli (Young’s modulus and shear wave velocity) was measured using SWE. The coefficient of variation values were calculated by using difference values of skin elastic moduli and skin thickness. A significant increase of both skin stiffness and thickness appeared in pre-treated keloids compared with post-treated keloids (P < 0.001) and normal controls (P < 0.001), respectively. Stiffness in post-treated keloids and normal skins was significantly different (P < 0.001), while the difference in thickness measurements showed no significance (P = 0.56, >0.05). The coefficient of variation of Young’s modulus was the highest when compared between (i) pre-treated keloids and theirs site-matched areas; (ii) pre-treated and post-treated keloids. SWE, which showed greater ability in determining the extent of keloids recovery, may provide an ideal tool to assess the stiffness of keloids and theirs therapeutic response.

## Introduction

**Figure 1:**
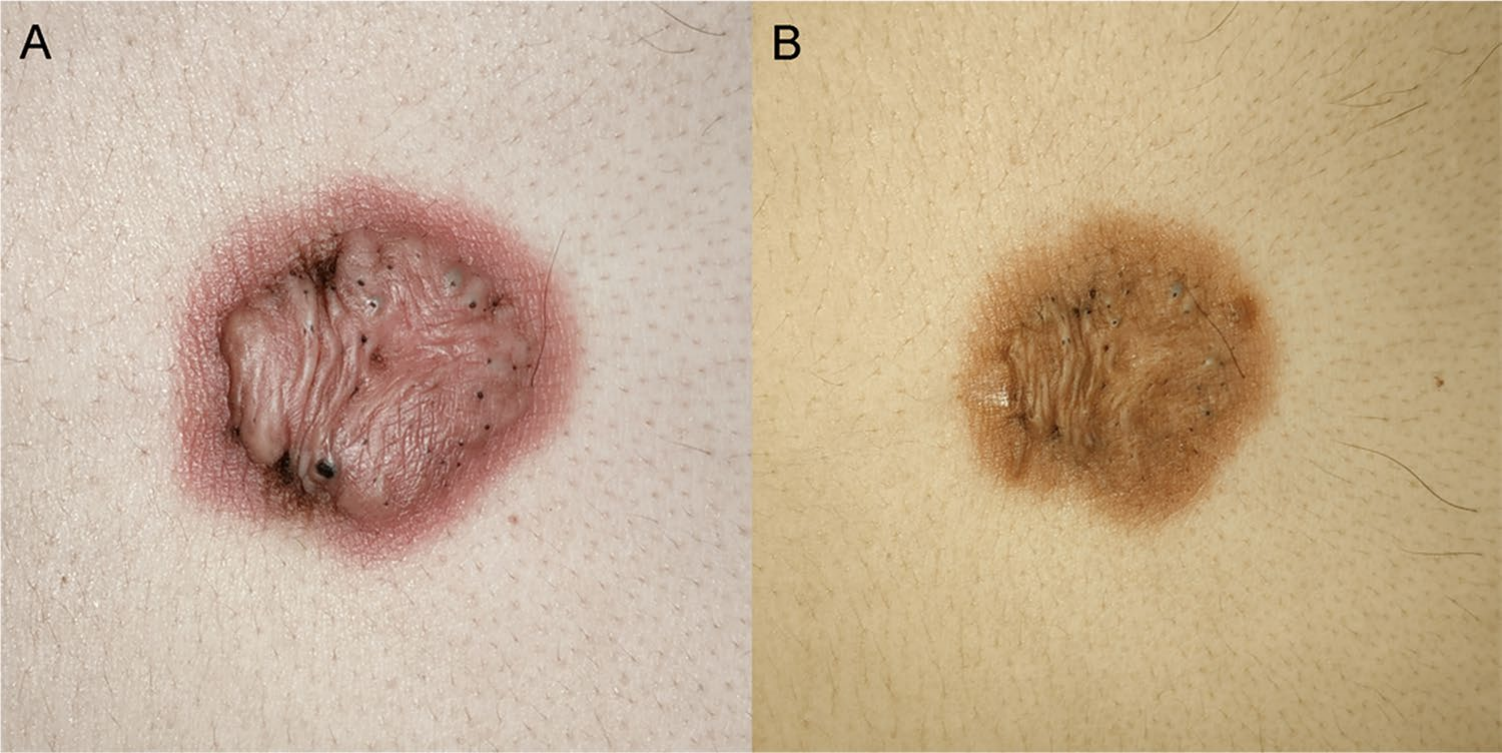
A Keloid in untreated condition (**A**) and treated condition (**B**). Image A and B correspond to the same patient, who received intralesional steriods injection.

Keloid, also known as keloid disorder and keloidal scar, is a type of pathological scar caused by incorrect regulation of the wound healing process beyond the original wound margin, which rarely regresses over time and may cause cosmetic disfigurement and physical impairment, ultimately leading to psychological stress^1–3^. Figure 1 showed a keloid on the chest wall of a male patient, which presented as a firm, pigmented, raised and untreated lesion (A) and a less raised injured tissue after intralesional steriods injection (B). As various therapeutic approaches for keloids were proposed to improve patients’ quality of life, the aim of evaluating keloidal scars and therapeutic effects has significantly attracted people’s attention.

Standardized questionnaires, such as the Patient and Observer Scar Assessment Scale, the Dermatology Life Quality Index, the Vancouver Scar Scale etc.^4–7^, are frequently used for clinical assessment of scars. However, it was reported that a gold standard rating scale for the assessment of scars has not been defined yet^8^, as data collection was dependent on clinicians’ and patients’ subjective observation and description. Moreover, the measurement of thickness using three-dimensional photography or negative–positive moulage is inaccurate as the portion of the scar below the skin surface is not included in the measurement^9^.

High-frequency ultrasound (HFUS), as a versatile diagnostic method with non-invasive and inexpensive properties, was introduced to evaluate keloids, which showed a great potential in assessment of thickness and echogenicity of keloid^3,7,9–11^. Another technique named ultrasound elastography, including strain elastography, acoustic radiation force impulse (ARFI) imaging, quantitative ARFI method and shear wave elastography (SWE)^12^, may assist observers in evaluation of the stiffness of keloids. Strain elastography provides a qualitative or semi-quantitative measurement of stiffness that is expressed as the ratio to a related area^13,14^ and ARFI imaging provides tissue displacement or related physical quantify within the ARFI push region^12^. By contrast, quantitative ARFI method and SWE have the advantage of providing quantitative data about elastic properties of tissue. Shear waves generated and propagated perpendicularly to axial displacement resulted from acoustic radiation force impulse or sonographic push pulses that emitted from transducer^13,15^. Measuring the shear wave velocity in a small region of interest (ROI) can be useful for the assessment of lesion stiffness. And according to the equation E = 3ρc^2^ (E the Young’s module, ρ the volume density of tissue and c the velocity), the Young’s modulus can be calculated simultaneously. Tissue elasticity is presented in terms of Young’s modulus (kPa) and shear wave velocity (m/s) in a real-time square color-coded ROI overlaid on gray-scale images^13,14,16^. To the best of our knowledge, strain elastography and quantitative ARFI method were both reported to be used in assessment of stiffness of keloids^15,17^. Moreover, the correlation between quantitative estimation of stiffness of keloid in quantitative ARFI method, clinical symptoms, and histopathological findings of the same keloidal lesions was previously demonstrated^15^. Nevertheless, only five cases were included in previous researches^15,17^. As for SWE, it has been reported to be used for detection of the stiffness of breast mass, thyroid, liver, prostate, nerve, and some other soft tissues^18–23^. It has also been demonstrated that SWE could be used for measurement of skin elasticity in recent years. For instance, a number of previous researches reported that SWE is feasible and reliable for measurement of normal skin elasticity^16^. For some skin diseases, such as systemic sclerosis, pathological changes in the disease may lead to changes in skin stiffness, which can also be measured and evaluated using SWE^7,24,25^. However, SWE and its availability in the assessment of therapeutic effect in keloid have not been tested yet. High quality researches regarding to SWE in the quantitative assessment of therapeutic response in keloids are required.

Thus, the aim of the present study was to measure skin thickness and elasticity in patients with keloids using HFUS and SWE respectively to indicate whether these two ultrasonic techniques could assess the therapeutic response in keloids.

## Methods

### Patients

From November 2017 to January 2019, 43 newly diagnosed patients (29 females and 14 males) with 76 keloids, were consecutively recruited at the Department of Ultrasound of West China Hospital of Sichuan University. The exclusion criteria were as follows: (i) Patients who received any treatment before; (ii) Patients with a history of metabolic disorders, endocrine diseases, rheumatic diseases, carcinoma or other dermatologic disorders except for keloids; (iii) Pregnant patients. The contralateral or surrounding skin of keloids, which was with no scars or any other pathological conditions, was regarded as the normal control site. Additional HFUS and SWE examinations were performed on these uninfluenced regions to obtain normal skin controls. Generally, keloid(s) and normal skin at corresponding site(s) in every patient were measured. When a patient has more than one keloid at the same site, there was only one normal skin control at the matched site, which corresponded to all keloidal scars growing at one body site. Imaging data of 76 keloids and 46 corresponding controls were obtained and analyzed after acquisition of informed consent from all the eligible patients. Besides, ethical approval was achieved from the West China Hospital of Sichuan University Ethics Committee. Table 1 shows the patients’ demographic and clinical characteristics.

**Table 1:** Patients’ demographic and clinical data.

Characteristics	Patients with keloids
Number of patients	43
Age (y)	
Mean ± SD (Range)	32.91 ± 13.58 (19–70)
Gender: Female/Male	29/14
BMI (kg/m^2^)	21.85 ± 3.12
Number of keloids	76
Specific locations:	
Chest wall	46
Shoulder	11
Perineum	6
Hip	5
Abdominal wall	4
Forearm	1
Umbilical region	1
Auricle	1
Neck	1
Number of skin controls	46

### Ultrasound and SWE examinations

**Figure 2:**
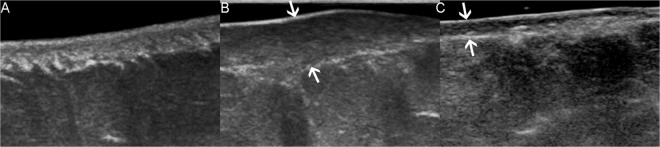
B-mode images (in longitudinal section) of normal skin (**A**), pre-treated keloid (**B**), and post-treated keloid accepted intralesional steriods injection (**C**). All images were captured from the same patient. Note the evident reduction of scar thickness after treatment, which was pointed by using arrows in image B and image C. Depth and width of images A-C were 1.5 cm × 2.25 cm, 1.5 cm × 2.25 cm and 1.6 cm × 2.4 cm, respectively.

The device used to obtain B-mode images of keloids and their corresponding normal skin controls and assess skin thickness was IU22 (Philips Healthcare, Amsterdam, the Netherlands). We used the L12-5 multi-frequency linear probe operating at 5–12 MHz to acquire B-mode images in this study, whose central frequency was 7.3 MHz. In addition, the mode was superficial musculoskeletal conditions. The tip of transducer covered with several millimeters of ultrasound gel was placed smoothly to the targeted skin area along perpendicular direction and in a longitudinal section. It was of great importance to ensure that there was no pressure between the probe and skin. Figure 2 illustrates three standard B-mode images of normal skin (A), pre-treated keloid (B), and post-treated keloid accepted intralesional steriods injection (C). Skin thickness expressed in millimeter was measured from the epidermis to dermis. For the normal skin, the epidermis and dermis exhibited on the screen were depicted as high-echogenecity and medium-echogenecity, respectively. Regarding the keloid, it appeared as lower echogenic zone on the screen and sometimes was displayed as inhomogeneous area.

**Figure 3:**
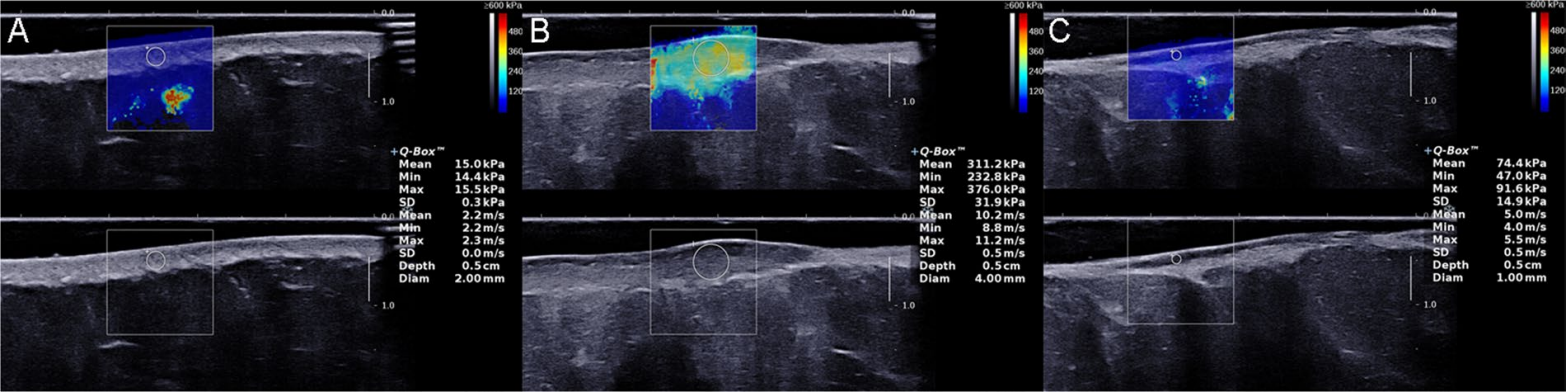
Elastography images of normal skin (**A**), and keloid in untreated condition (**B**) and treated condition (**C**). The post-treated keloid was accepted intralesional steriods injection and all images were collected from the same patient as in Fig. 2. The selected quantitative values, including the mean values of Young’s modulus and shear wave velocity were automatically displayed on the image. The decrease of the two values after therapy is clearly visible.

SWE examination was undertaken by a trained sonographer using Aixplorer US system (SuperSonic Imaging, Aix-en-Provence, France) with a SL 15–4 linear probe operating at 4–15 MHz (central frequency: 7.5 MHz). And the superficial musculoskeletal mode was preset. The precautions of manipulating the probe were as same as when acquiring a B-mode image by using another device. After switching to SWE-mode, the operator maintained the transducer for few seconds to obtain a stable color-coded SWE image. Ultrasound gel covering on the probe tip was proven not to influence the appearance of the elastogram^16,26^. The square region of interest, which was marked on targeted skin area, was used to collect SWE data. Colors, as shown in the ROI, varied along with the changing the skin stiffness. A very soft tissue was coded by dark blue and a very hard lesion was coded by red, while other colors (light blue, green, and orange) represented the increase of stiffness of tissue between very soft tissue and very hard lesion. After acquisition of a stable color-coded square ROI, skin elasticity was measured in a small round area called Q-box, which was put in the middle of the ROI. The diameter of Q-box varied due to the size of targeted zone. A series of the elastic moduli, including the mean, maximum, minimum, and standard deviation for values of both Young’s modulus and shear wave velocity were calculated by the system and exhibited on the right side of the screen automatically. Figure 3 exhibits three standard elastograpy images of normal skin (A), and keloid in untreated condition (B) and treated condition (C), which were collected from one patient who was accepted intralesional steriods injection. Three measurements, which meant three Q-boxes in three images, were performed at each site. The mean values of Young’s modulus and shear wave velocity were selected as representative values for each obtained image in this study. Additionally, the averaged mean Young’s modulus value (E-mean) and the averaged mean shear wave velocity value (C-mean) were presented as kPa and m/s, respectively.

### Study protocol

The ultrasound examination was performed on all the patients by an experienced sonographer who received SWE training. Each participant in this study received both HFUS and SWE examinations for two times. All the patients had never been treated before their first examination and were recalled to be examined again after receiving different therapies. There were six different therapeutic regimens applied appropriately on 76 keloids according to patients’ conditions. The majority of keloids received intralesional steroid injection (64 keloids), while the other 12 keloids were treated by radiation therapy (4 keloids), occlusive dressings (3), surgical excision of scarring lesion (1), combination of radiation therapy and laser therapy (2), and combination of injection and radiation therapy (2). The interval of different patients between the first-time examination and reexamination was different in the present study. And the interval was no longer than 11 months. Examining items were set as follows: (i) ultrasound at B-mode (including measurement of skin thickness) and SWE for evaluation of both keloidal lesions and corresponding normal controls were performed in the first-time examination. (ii) During reexamination, identical ultrasonic evaluating items were only applied again on keloids.

### Statistical analysis

In this study, SPSS 20.0 software (IBM, Armonk, NY, USA) was used to carry out statistical analysis; P < 0.05 was the two-side significance level. The comparison of skin thickness and skin stiffness among normal skin controls (NS group), pre-treated keloids (UNTK group), and treated keloids (TK group) was undertaken using Friedman test. Pairwise comparison and descriptive statistics expressed as median were performed for acquisition of further specific outcomes. We calculated the differences in skin thickness between UNTK group and TK group, and expressed them as mean ± standard deviation (SD). Moreover, we applied the same calculating process for skin elasticity, including both E-mean and C-mean. We also made an identical comparison between UNTK group and NS group. Calculation of the coefficient of variation (CV), which is defined as the ratio of the standard deviation to the mean, of these differences helped find out which index was more sensitive for identifying keloids and treatment efficacy.

## Results

### Comparing skin thickness among UNTK group, TK group, and NS group

**Figure 4:**
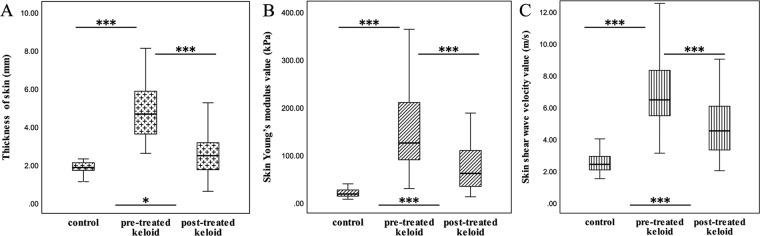
Box-and-whisker plots of skin thickness (**A**), E-mean (**B**) and C-mean (**C**) in NS group (skin controls), UNTK group (pre-treated keloids), and TK group (post-treated keloids). The top and bottom of each box represent the 75th and 25th percentiles, respectively; the median, the minimum, and maximum are represented by the horizontal line, as well as the top and bottom of the whiskers in each box, respectively. All pairs of compared groups showed a significant difference except that no statistical significance was found in skin thickness between NS group and TK group (P = 0.56). ***P < 0.001, *P > 0.05. E-mean = averaged mean Young’s modulus; C-mean = averaged mean shear wave velocity.

Table 2 shows the descriptive statistical data, which were expressed as the median of the skin thickness in each group. Skin thickness was significantly higher in UNTK group than that in NS group (P < 0.001) and TK group (P < 0.001). The differences in skin thickness between TK group and NS group were not statistically significant (P = 0.56, >0.05) (Fig. 4A).

**Table 2:** The median of skin thickness measured by HFUS and two values related to skin elasticity were assessed by SWE in each group, in addition to the comparison of each parameter between any two groups.

Parameters	Groups			P
	UNTK	TK	NS	
H (mm)	4.65^†^	2.47	1.85	P < 0.001^a,b^
E-mean (kPa)	124.60	60.85	17.70	P < 0.001^a,b,c^
C-mean (m/s)	6.45	4.50	2.40	P < 0.001^a,b,c^

^†^Median.

H = skin thickness; E-mean = averaged mean Young’s modulus; C-mean = averaged mean shear wave velocity; UNTK = pre-treated keloids; TK = post-treated keloids; NS = normal skin controls

mm: millimeter; kPa: kilopascal; ^a^UNTK group versus TK group; ^b^UNTK group versus NS group; ^c^TK group versus NS group.

### Comparing quantitative SWE features among UNTK group, TK group, and NS group

Table 2 lists the median of skin stiffness represented by Young’s modulus and shear wave velocity in each group. Quantitative SWE features, including both E-mean and C-mean, were higher in UNTK group and TK group than those in NS group, and were the highest in UNTK group (P < 0.001 for all) (Fig. 4B,C).

### Comparing three different parameters

The CV values of differences are shown in Tables 3 and 4. CV_E_, CV_V_, and CV_H_ were abbreviations for CV values of differences in E-mean, C-mean, and skin thickness, respectively. For making comparison between UNTK group and TK group, CV_E1_ was maximum among CV_E1_, CV_V1_ and CV_H1_ (Table 3). When a comparison was made between UNTK group and NS group, the maximum value among CV_E2_, CV_V2_, and CV_H2_ was CV_E2_ (Table 4). And CV_E1_ was maximum among all six CV values (Tables 3 and 4).

**Table 3:** Difference values in three parameters (skin thickness, mean Young’s modulus, and mean shear wave velocity) between UNTK group and TK group and three CV values were calculated by using difference values.

Parameters	Difference value (Mean ± SD)	CV (%)
Skin thickness (mm)	2.43 ± 2.08	85.53
E-mean (kPa)	69.83 ± 70.18	100.49
C-mean (m/s)	1.93 ± 1.77	91.56

**Table 4:** Difference values in three parameters (skin thickness, mean Young’s modulus, and mean shear wave velocity) between UNTK group and NS group and three CV values were calculated by using difference values.

Parameters	Difference value (Mean ± SD)	CV (%)
Skin thickness (mm)	3.08 ± 1.99	64.58
E-mean (kPa)	126.42 ± 84.76	67.04
C-mean (m/s)	4.12 ± 1.93	46.85

E-mean = averaged mean Young’s modulus; C-mean = averaged mean shear wave velocity; CV = coefficient of variation (defined as the ratio of the standard deviation to the mean).

CV_H1_ = 85.53%; CV_E1_ = 100.49%; CV_V1_ = 91.56%.

CV_H2_ = 64.58%; CV_E2_ = 67.04%; CV_V2_ = 46.85%.

## Discussion

HFUS is generally used to assess skin echogenic changes and can also provide thickness of keloids^3,9–11,27^. In the present study, the thickness of keloids before treatment was significantly thicker than that of normal skin (P < 0.001). Moreover, the skin thickness in keloids was significantly changed before and after treatment (P < 0.001). These results revealed that HFUS could help differentiate keloids from normal skin, and also assess thickness of keloids before and after treatment, which is consistent with previous findings^3,9–11,27^. It should be noted that in the present study, the difference in skin thickness between TK group and NS group was not statistically significant (P = 0.56, >0.05), indicating that the skin thickness in keloids basically reduced to the normal level after treatment (Fig. 4A).

The present study focused on applying SWE to measure the skin stiffness of keloids, and found that the skin elasticity expressed in E-mean and C-mean significantly differed between UNTK group and TK group, and also between normal skin and keloids in both UNTK group and TK group (Figs. 4B,C). The findings demonstrated that the measurement of the Young’s modulus and the shear wave velocity showed a great capability in differentiating normal skin from keloids. For keloids, the capacity of these two parameters to differentiate untreated cases from treated cases was also found. According to a more detailed analysis of the result in this study, we found that although the skin stiffness after treatment was lower than that before treatment, difference in stiffness between post-treated keloids and the normal skin could be still observed. Nevertheless, this statistical result was partly different from that of skin thickness, which meant that the skin thickness at the treated keloid was almost as same as normal skin thickness. It showed that the Young’s modulus and the shear wave velocity were values that may detect the difference between normal skin and post-treated keloids, but the difference in skin thickness between the two was not statistically significant, which indirectly verified the superior performance of skin elastic modulus in detecting keloids compared with skin thickness.

Quantitative measurements of skin thickness, E-mean, and C-mean were all suggested to be effectively used to evaluate post-treatment efficacy based on the above-mentioned results. In order to compare the sensitivity of these three indicators to assess the therapeutic efficacy, we separately calculated the differences among the three sets of data (skin thickness, E-mean, and C-mean) in UNTK group, and the corresponding data between TK group and NS group. Six CV values (CV_E1_, CV_V1_, CV_H1_, CV_E2_, CV_V2_, and CV_H2_) were obtained, which were divided into two groups (see Tables 3 and 4) According to the result of comparison between these two groups, the variation of the Young’s modulus value was the highest, whether it was compared between the pre-treated and post-treated keloids, or between the keloids before treatment and normal skin. It is noteworthy that the larger the CV value is, the better ability of differencing between normal skin and keloids in untreated and treated conditions, as well as discriminating therapeutic effects of the parameter represented by the CV value is proven to have. Therefore, the outcomes indicate that the Young’s modulus was the strongest indicator for comparing normal skin and keloids, as well as identifying the effects of keloid treatment.

A number of unique features of the present study should be pointed out. Firstly, to our knowledge, this was the first study concentrated on the feasibility of SWE to indicate the stiffness of keloids, and compare the differences before and after treatment. Secondly, the present study, for the first time, indicated whether skin thickness or skin stiffness expressed as values of Young’s modulus and shear wave velocity has a stronger ability to discriminate the therapeutic effects. The above-mentioned findings revealed that SWE could provide a potential measure for evaluating keloids. However, to further express the ability of SWE in assessment of keloids and the estimation of treatment responses, a correlation analysis using the results of clinically used evaluation methods should be investigated. There are a number of limitations in the current research. The number of samples in this study is still relatively small. Moreover, because this study is a preliminary study, keloids that received different treatments were not grouped to compare the efficacy of applied treatments. These aspects need to be further refined in the future.

In conclusion, the current research suggested that in addition to HFUS, SWE could also be used to quantitatively differentiate keloids from normal skin. And SWE showed a better ability to accomplish this task. In terms of evaluating the efficacy of treating keloids, the method of measuring skin stiffness using SWE is more sensitive than the method of measuring thickness using HFUS. The results indicated that SWE, a new, quantitative, and non-invasive method, has a great potential for evaluation of the therapeutic response in keloids. In the future research, we will attempt to increase sample size and further validate the role of SWE in identifying keloids, monitoring changes in keloids before and after treatment, as well as assisting clinicians to investigate the curative effect of different therapeutic methods.

## Data Availability

The datasets generated during and/or analysed during the current study are available from the corresponding author on reasonable request.
